# New medical schools in Sub-Saharan Africa –a cross-sectional survey of educational structures, operations, and policies

**DOI:** 10.3389/feduc.2023.1232822

**Published:** 2023-12-11

**Authors:** Krish Shah, Elizabeth S. Rose, Andrew Rees, Seun Falayi, Quentin Eichbaum

**Affiliations:** 1 Department of Psychology and Human Development, Peabody College, Vanderbilt University, Nashville, TN, United States,; 2 Vanderbilt University School of Medicine, Vanderbilt University Medical Center, Nashville, TN, United States,; 3 Atrium Health, Charlotte, NC, United States,; 4 University of Ibadan, Ibadan, Nigeria

**Keywords:** medical education, Sub-Saharan Africa, pedagogy, curriculum development, global health

## Abstract

**Introduction:**

Africa does not have enough doctors despite having the highest continental burden of disease. Encouragingly, many new medical schools are opening and have begun to graduate doctors. However, the educational structures, operations, and policies of these schools remain poorly understood. This study aimed to better understand these dimensions of new medical schools on the continent.

**Methodology:**

We developed and implemented an online survey covering topics that included admissions policies, curricular design, assessment, accreditation, faculty development, research capacity, postgraduate training, and COVID-19-specific challenges. The survey was sent to 130 schools of which 52 represented individually identifiable email addresses (the remainder being schools’ websites or generic addresses).

**Results:**

Responses represented 10 countries (response rate ~ 56%). Curricula were mostly lecture-based (*n* = 18, 75%). Electronic platforms and information technologies were used by over 75% (*n* = 18) of schools. More than half have not implemented postgraduate training programs (*n* = 13, 57%). Most schools had a formal accreditation process (*n* = 16, 70%), but the source of accreditation varied. The biggest challenge facing schools was financial (*n* = 20, 87%) followed by faculty/staff recruitment, training, and retention (each *n* = 15, 65%).

**Conclusion:**

New medical schools in Sub-Saharan Africa are a gateway to the next generation of medical doctors in a region where medical professionals are desperately needed. This survey of new schools is an important step in better understanding their status and needs, especially with the onset of the pandemic.

## 1 Introduction

An oft-quoted statistic about health care in Africa is that the continent accounts for 23% of the global burden of disease but harbors only about 3% of the global healthcare workforce (Anyangwe and Mtonga, 2007; Global Burden of Disease Collaborative Network, 2021). How to increase the size of the workforce and adequately capacitate health care on the continent remains an immense and unsolved problem.

A direct approach would be to simply train more doctors and nurses. Additional health professionals are sorely needed especially in Sub-Saharan Africa (SSA) (Frenk et al., 2010; Tsinuel et al., 2016). This training could be achieved either by increasing the intake of students at existing medical schools or by building new schools. Given the large numbers of doctors and nurses needed, existing schools could likely not manage an increased intake. Whereas high-income countries, like the United States of America, have more than 6 medical schools per 10 million people to train health professionals, most countries in Africa have fewer than half that number (Frenk et al., 2010). Establishing new medical schools is a complex challenge especially in low-resource rural settings where they are most needed and where essential associated facilities are limited (Tsinuel et al., 2016; Eichbaum, 2017).

The increased number of new medical schools opening on the African continent is a welcome development (Greysen et al., 2011). Whether all these schools will ultimately succeed in producing doctors remains to be seen. Such well-intentioned initiatives are often constrained by political will, national budgets, and lack of well-trained faculty.

Gaining a better understanding into how current new schools operate and are performing could provide insights for planning new schools. To date however, no such study has been published. New medical schools encounter a range of challenges, including scarce financial resources, infrastructure challenges, faculty, and staff retention issues, and initially low numbers of graduates (Mullan et al., 2011; Eichbaum et al., 2012; Tsinuel et al., 2016). The 2010 Lancet report on global health professional education argued that new medical schools could be nimbler and more innovative than established schools that are often encumbered by “curricula rigidities, professional silos, static pedagogy, (and) insufficient adaptation to local contexts” (Frenk et al., 2010, p. 1,926). New medical schools have the potential to leapfrog over traditional educational models toward creative educational alternatives (Frenk et al., 2010; Eichbaum et al., 2015). Among the innovations considered by new medical schools are technological innovations, new models of assessment, and incorporation of community service-based learning. Yet the extent to which such innovations have been successfully and sustainably implemented has been less examined (Greysen et al., 2011; Mullan et al., 2011; Frambach et al., 2017).

Medical curricula should be contextualized to local and regional healthcare needs (Bleakley et al., 2008; Eichbaum et al., 2015; Tsinuel et al., 2016). Modern medical education is shifting from traditional teaching and assessment methods to learner-centered methods including competency-based curricula, problem-based learning, community experiences, and e-learning, among others (Mokone et al., 2014; Eichbaum et al., 2015; Tsinuel et al., 2016; Barteit et al., 2019). Innovative electronic tools and resources have been proposed to make anatomy more inviting and engaging in SSA through the use of educational games, videos, online sources, and 3D printing (Gbolahan Balogun, 2019). In Tanzania, the adoption of technology-enhanced learning has resulted in increased acceptance of blended learning programs, increased accessibility of learning opportunities, improvements in in-person instruction, and strengthening of international relationships (Mtebe and Raphael, 2017). However, the researchers note that the major limitations for blended learning with technology are infrastructure-related, such as shortages in electricity, internet bandwidth, funding, and government policymaking.

Ideally, establishment of a medical school will be followed by accreditation from a recognized organization (Tsinuel et al., 2016), generally with the support of the national government. The World Federation of Medical Education (WFME) is a global organization established to enhance the quality of medical education worldwide and it certifies medical education accreditors.^1^ Although SSA has one of the oldest medical education accreditors in the world, the National Universities Commission of Nigeria, only one accreditor, the Sudan Medical Council, is recognized by the WFME and 48% of countries do not yet have accreditation for undergraduate medical education (Bedoll et al., 2021).

New schools can be innovative regarding student admission policies. In a review of medical school global admissions policies, researchers at Mulungushi University in Zambia suggested that schools in Africa should consider academic as well as non-academic elements in medical school admissions (Ezeala et al., 2020). While academic excellence may predict achievement in the pre-clinical stage, non-academic attributes were reported to be predictive of success in the clinical stages of training (Ezeala et al., 2020). Using selection instruments based on a variety of factors, including socioeconomic status, can help select the most fitting candidates and fulfill a school’s social obligation to include students from a variety of backgrounds. Wilson et al. (2009) proposed that to redress the imbalance of urban versus rural medical trainees, new medical schools and satellite campuses should be opened in rural areas. Since trainees from rural areas are more likely to return to work in those areas, an admissions quota could be established for candidates from rural areas (as has been done at the University of Namibia School of Medicine) and preference might be given to candidates intending to practice in rural areas.

In light of the recent COVID-19 pandemic, many academic global health programs, especially in low-and middle-income countries, suffered from reduced scholarly output, education program funding, and negative consequences on research due to disruptions in communication and international travel (Rose et al., 2022). Adaptations and transitions to virtual learning occurred world-wide. Bernard et al. (2021) found that 80% of medical schools in Africa suspended classes due to the COVID-19 pandemic and 59% remained closed months after the suspension. Of the students who resumed classes, 70% reported participating in online classes, 19% hybrid, and 17% in-person. Specifically, in Nigeria transitions to e-learning resulted in little to no effect for students in their preclinical years, but clinical exposure was greatly impacted resulting in the loss of valuable time and a delay in matriculation for health care professionals (Oladipo et al., 2020).

A survey of medical schools in SSA was last conducted in 2012 and provided aggregated information about well-established and newly established schools (Chen et al., 2012). Although some newer medical schools have described their experiences (Mokone et al., 2014), relatively little is known about new schools, especially ones established in the past decade.

The aim of this study was to gain insight into education structures, operations, policies, successes, and challenges of new medical schools established in SSA in the last two decades. A carefully developed web survey was used to gather qualitative and quantitative information about these schools’ admissions policies, curricula, preclinical and clinical assessment, evaluation and accreditation processes, research capacity, postgraduate training programs, and faculty development, as well as issues that face these schools and their operating capacities. This article reports on the findings of that survey.

## 2 Method

Working with colleagues in Africa and Europe, we developed a comprehensive survey in English that covered topics including demographics, funding and partnerships, admissions, curricular structure and resources, postgraduate programs, accreditation, and overall challenges. We developed the survey in Research Data Capture (REDCap), a secure web-based platform for survey development and data storage (Harris et al., 2009, 2019).

Survey question types included multiple choice (single and multiple response), binary measures (yes/no), Likert scales, and comment boxes. The dichotomized questions in the survey asked about whether the medical school had satellite campuses, classes were held during the pandemic, and about plans to resume in-person classes. The two questions in the survey with Likert scales asked about the average age of the admitted student body and percent of the student body that is female. We pilot tested the survey at three new medical schools in SSA with educators who were not respondents in the final survey. We then emailed the online survey and an explanatory cover letter to prospective participants between February 2021 and April 2021. Reminders to complete the survey were emailed a week later and there was no coercion for completion. Prospective participants were deans or other faculty representatives of medical schools in SSA that started admitting students after 1999. We identified these schools and contacts from the WFME website and the Consortium of New South African Medical Schools (CONSAMS).

We collected and stored data in REDCap before exporting for analysis in JASP, Excel, and SPSS. We calculated descriptive statistics, including means and standard deviations, based on the total number of responses for each question. We conducted correlation tests for selected questions based on research from our literature review. We generated graphs, tables, and figures using Tableau.

Ethics approval for this study was received from the Vanderbilt University Medical Center Institutional Review Board (IRB). Participants were assured of anonymity and confidentiality in their responses, and they consented to participate in the study.

## 3 Results

### 3.1 Quantitative data

#### 3.1.1 Demographics

We identified 130 new medical schools in name including schools in Portuguese-and French-speaking counties. Obtaining the individual contact information for more reliable responses from each school was problematic. Despite considerable effort, we managed to identify personal contacts and email addresses at only 52 of these schools. For the remaining schools we had only generic addresses (such info@--, dean.med@--, registrar@--, and faculty@--). Given the high number of “undeliverable” responses we received, coupled with a high number that likely went to spam or were ignored, the total response rate (out of 130) was artificially depressed to 29%. However, if we count responses only from those sent to individual email contacts, the response rate was 56%. We argue that the response rate of 56% is more accurate.

Ten countries were represented in the survey responses and most individual respondents were deans at their respective school (*n* = 16, 64%). While the median year that schools began admitting students was 2012, 80% (*n* = 20) were established in 2010 or later (Figure 1). Many institutions were in metropolitan areas (*n* = 17, 68%), while the remainder were distributed in towns (*n* = 7, 28%) and rural areas (*n* = 1, 4%) (Table 1). Some schools had satellite campuses (*n* = 6, 24%). Although the mean gender distribution of school populations was even (*F* = 49%, *n* = 11; M = 51%, *n* = 13), there was significant variance between schools (standard deviation = 16%).

#### 3.1.2 Funding and partnerships

Most institutions were either public (*n* = 11, 44%) or private (*n* = 9, 36%), while a few represented public-private partnerships (*n* = 4, 16%). Funding for medical schools was primarily from government (*n* = 11, 44%) or private funds (*n* = 11, 44%). Some schools were funded by philanthropic donations (*n* = 2, 8%) and parastatal funds (*n* = 1, 4%). Nearly all (*n* = 21, 91%) reported that their school had formal agreements/working relationships with other universities/training programs. Those relationships were with universities and programs that were within their country (*n* = 16, 76%), within Africa (*n* = 14, 67%), and/or outside of Africa (*n* = 13, 62%).

#### 3.1.3 Admissions

Decisions for student admission were made largely by the medical schools’ admissions committee with minimal government input or quotas (*n* = 16, 67%). Most admissions committees were composed of clinical faculty (*n* = 15, 63%). All schools indicated using high school or other entrance exams to determine admission (*n* = 24). A small percentage used interviews (*n* = 7, 29%), holistic qualities (*n* = 4, 17%), or extracurricular activities (*n* = 2, 8%) in addition to high school or entrance examinations.

#### 3.1.4 Curricular structure and resources

All schools had programs that were five to seven years in length, with a six-year degree program the most common (*n* = 15, 63%). The type of medical degree offered was nearly equal between Bachelor of Medicine, Bachelor of Surgery (MBBS) (*n* = 7, 28%); Bachelor of Medicine and Bachelor of Surgery (MBChB) (*n* = 7, 28%); and Doctor of Medicine (MD) (*n* = 10, 40%). Most schools developed their own curriculum (*n* = 18, 75%) but 21% (*n* = 5) used a curriculum donated from another source (Table 2). Nearly all respondents agreed or strongly agreed that their curriculum was sufficiently contextualized to local needs (*n* = 24). Half of schools used a curriculum with a “traditional” program of two to three years of biomedical science followed by two to three years of clinical training (*n* = 12). Medical schools used various combinations of learning philosophies and methods, including lecture- (*n* = 18, 75%), case- (*n* = 15, 63%), and/or problem-based learning (*n* = 15, 63%). Most schools conducted clinical rotations in the same locale as the medical school (*n* = 16, 67%) and/or in the surrounding health care facilities (*n* = 18, 75%).

Almost all schools incorporated research or research methodologies into the curriculum (*n* = 22, 96%). The research courses included classes on research design (*n* = 23, 100%), quantitative analysis (*n* = 21, 91%), qualitative analysis (*n* = 20, 87%), and ethics (*n* = 17, 74%). Research included in the formal curriculum focused on public/population health (*n* = 18, 78%), clinical (*n* = 17, 74%), and biomedical science (*n* = 14, 61%). Anatomy courses varied in how they were conducted. Many medical schools used synthetic, anatomical models (*n* = 15, 63%) or student participation in dissection of cadavers (*n* = 13, 54%). All schools used multiple methods of anatomy education.

Students’ access to paid, online learning resources, like ScholarRx or Lecturio, was rare (*n* = 4, 17%). Most schools (*n* = 19, 79%) included free study resources, like Khan Academy and Coursera, in the curriculum. Many students regularly used an information technology library (*n* = 20, 83%), laptops/tablets (*n* = 18, 75%), and/or smart phone applications (*n* = 15, 63%) for coursework. Over half of schools used a learning management system, such as Moodle or Blackboard (*n* = 13, 54%). Assessment during the preclinical phase included multiple-choice (*n* = 24, 100%), written essay (*n* = 21, 88%), and/or oral (*n* = 17, 71%) components. Students were assessed similarly in the clinical phase; however, practical assessments (*n* = 21, 88%) were also included.

#### 3.1.5 Postgraduate programs

The schools’ postgraduate programs offered either few specialties or specialties distinct from the survey response options as many respondents indicated that “none” of the listed specialties were offered (*n* = 13, 57%). However, the most commonly selected specialties of those listed were Surgery (*n* = 6, 26%), Obstetrics and Gynecology (*n* = 6, 26%), and Internal Medicine (*n* = 4, 17%).

#### 3.1.6 Accreditation

Most schools (*n* = 16, 70%) implemented a process of formal accreditation. Of those schools, most were accredited by a national accreditation body (*n* = 21, 91%) and/or an internal quality assurance office/committee (*n* = 13, 57%). Less than half (*n* = 11, 48%) reported that their accreditation guidelines aligned with those set forth by the WFME, while 52% (*n* = 12) did not know if their guidelines aligned with the WFME.

#### 3.1.7 Challenges

The biggest challenges schools faced included financial issues (*n* = 20, 87%), recruitment and retention of faculty/staff (*n* = 15, 65%), and faculty/staff training and professional development (*n* = 15, 65%) (Table 3). In light of the COVID-19 pandemic, financial troubles were exacerbated for most medical schools (*n* = 19, 83%). All but one school still held classes during the pandemic. Many schools (*n* = 16, 70%) held a combination of in-person and online courses, but 22% (*n* = 5) held solely in-person courses and 9% (*n* = 2) were solely online.

We looked for correlation between a variety of dimensions including school demographics, curricular design, accreditation, and challenges but found no significant correlations. The diversity among schools in structures, operations and policies in different countries couples with the relatively small number of schools may have contributed to statistically insignificant associations.

### 3.2 Qualitative data

In open-ended response questions, we asked about strengths of schools and innovations that the school developed to address challenges. Five themes emerged: management, workforce, infrastructure and resources, curriculum, and partnerships. Respondents described features of strong management practices including teamwork, self-reliance, openness to discussions, transparency, and flexibility. Outputs of their school’s work included having a “good reputation in the society,” engaging with the community, and improving quality education.

Respondents described their faculty and staff as “passionate,” “energetic,” “well-experienced,” and “dedicated.” From these descriptions, it was apparent that respondents were enthusiastic about their workforce. However, respondents mentioned challenges in building their workforce. Some wrote about overcoming this challenge through utilizing “part-time lecturers from other faculties,” “virtual lecturers from other schools,” and providing training. Others were challenged by part-time, young, or inexperienced faculty as well as low qualifications of lecturers and specialized faculty members.

While some respondents wrote positively about school facilities including an affiliated clinic, laboratories, a library, and lecture rooms, many others described a lack of infrastructure, laboratory equipment, and clinical rotations as well as financial limitations that prevented expansion. One respondent described innovatively “owning our teaching hospital rather than using regional hospitals,” which avoided some resource and rotation challenges.

Curriculum was also a common theme among innovations and strengths. Examples included “strong assessment procedures,” “curriculum review and development of short courses,” and “using a new and different curriculum” than is used nationally.

Other innovations and strengths included building partnerships and signing agreements with other institutions. These partnerships spanned national, international, government, non-governmental, and Christian organizations.

## 4 Discussion and conclusion

The number of new medical schools in Africa has been rapidly increasing especially in the last decade (Figure 1) (Tsinuel et al., 2016). Although the sample studied in this survey revealed many differences between schools regarding the selected parameters, there were also similarities.

Compelling arguments have been advanced in favor of establishing new medical schools and training hospitals in rural communities because rural areas are where healthcare needs are acute and unmet. Training doctors in these areas could help meet these needs (Wilson et al., 2009; Eichbaum, 2017). Although most schools in our study were in metropolitan areas, a quarter of schools utilized clinical placements in rural areas and one school had distributed campus locations in ten districts and regions. Rural initiatives are known to impact students’ decisions to practice in rural areas after graduation (Eichbaum, 2017; Szafran et al., 2020). To increase equity among applicants from rural and urban areas, some schools have used holistic admission criteria decisions (Wilson et al., 2009; Eichbaum et al., 2015), as did a few in our study.

There were similar proportions of public and private schools, indicating that the number of private schools is increasing as there were fewer private schools established in the earlier years of our time range. This increase might be driven largely by the urgent need to graduate more doctors in these countries. Accreditation of such schools confers legitimacy and affirms their ability to produce high quality graduates. Additionally, private schools can operate with more autonomy and less government interference (Mokone et al., 2014; Tsinuel et al., 2016). Public-private partnerships have been offered as a solution (Tsinuel et al., 2016). A small percentage of the schools in this study represented public-private partnerships and notably all such schools were established in the last decade, suggesting a trend toward these partnerships.

Accreditation is a vital step for schools. It is concerning that not all the surveyed schools had implemented a process for accreditation and over half did not know if their school was aligned with accreditation guidelines from the WFME. Challenges arise in balancing accreditation standards and tailoring education to meet local needs (Frenk et al., 2010; Eichbaum et al., 2015). Although WFME accreditation provides schools an opportunity for global accreditation, it was developed by Europeans and has been viewed by some as a perpetuation of colonialism and penetration of Western ideas into African education (Weisz and Nannestad, 2021). Critics note that while some accreditors encourage incorporating local perspectives, others continue to promote Western standards for core competencies (Bleakley et al., 2008). Colonialist arguments have also been made against problem-based learning, a popular teaching method and a key component of some accreditation standards (Frambach et al., 2017) and characteristic of some surveyed schools’ curriculum.

All schools surveyed had a five-to seven-year curriculum, reminiscent of colonial era curriculum. Respondents noted, however, that their school’s curriculum was contextualized to their region despite a quarter of curricula being donated from elsewhere. Contextualization helps ensure that students learn about diseases, diagnoses, and treatments that are regionally and culturally relevant (Frenk et al., 2010). Despite the most common teaching method being teacher-centered and lecture-based, student-centered approaches were used in over half of the schools. Of the schools that utilized student-centered designs, most were established in the past decade, perhaps reflecting current trends toward these teaching methods as well as the innovative capacity of these newer schools. Additionally, it was more common for the newer schools to integrate biomedical science and clinical training throughout the curriculum, which is an innovative curricular approach and further suggests a trend of newer schools toward innovation (Dahlman et al., 2018).

E-learning and telehealth provide potential solutions to training medical professionals and mitigating the impact of health worker shortages. Prior to the COVID-19 pandemic, e-learning was underdeveloped globally and particularly in SSA, needed more funding and needed to be implemented with a systems-wide approach for sustainability (Barteit et al., 2019). Most respondents reported that their schools utilized free online resources and students used commonly available technology equipment (e.g., tablets, smart phone applications, etc.) to complete coursework, however this is not the case for all higher education institutions in Africa. Among higher education training institutions in Africa, only 38.5% note adequate access to e-learning platforms during the pandemic (Ossai and Ogbuoji, 2021). In terms of research and faculty, budget cuts and reallocation of research funding will lead to decreased diversification in research proposals and overall, less research by faculty in Africa (Ossai and Ogbuoji, 2021).

Finances were a common challenge of schools, which likely prevented investment in paid educational resources and equipment to support learning. Additionally, infrastructure can be a challenge, as was described by Walsh et al. (2018) in implementing e-learning in Liberia. When impelled to utilize online resources and technologies due to the pandemic, almost 80% of surveyed schools offered either partial or full course loads online. The COVID-19 pandemic has been reported to have caused an abrupt transition from in-person community-based learning to online delivery of course content (Goh and Sandars, 2020; McKimm et al., 2020).

The common postgraduate program specialties that were offered by schools in our study (i.e., surgery, obstetrics/gynecology, internal medicine, pediatrics, and occupational/public health) differed from those described by other authors. Talib et al. (2019) reviewed 813 publications from 2005 to 2016 on postgraduate training in SSA and found surgery, anesthesiology, emergency medicine, and family medicine specialties to be the most common. In addition to different specialties, over half of our respondents marked the answer option “none” in our list of specialties. Since many of the schools were established within the past decade, perhaps they had not yet established postgraduate programs. It is also possible that these schools have opted to focus on undergraduate medical training rather than postgraduate training.

These deeper understandings about curricular elements, accreditation, challenges, and innovations provide valuable insight to SSA medical schools. These findings could be used to strengthen current schools and plan effectively for schools that will train the next generation of medical professionals.

### 4.1 Limitations

This study had limitations. Despite considerable effort, we were able to obtain individually identifiable contact information for only 52 of the 130 medical schools so that the majority of the surveys were sent to generic addresses and websites. The intention of our strategy of sending the survey to all addressed was to obtain more responses - but most returned as undeliverable or did not get a response. This limiting factor artificially depressed the total response rate to 29% but counting responses sent to identifiable addresses the (likely more accurate) response rate was 56%. The range of developmental stages of new medical schools and their staffing shortages likely also contributed to the difficulty in finding reliable contacts; and the WFME site from which much of the list was compiled provided minimal identifiable contact information and mostly generic contact information.

We conducted the survey in English and therefore may have unintentionally excluded the Francophone schools of West African and Lusophone schools, especially from Mozambique and Angola where a considerable number of new medical schools have been in development. However, one school each from Mozambique and Francophone Cameroon responded to the survey.

We are unsure if the individual responding to the survey was the most appropriate person from the school to provide information. The accuracy of their responses could not be verified and may have resulted in some unreliable responses.

To maintain a reasonable survey length, we were not able to query schools on all components of their curriculum or school design. For example, we did not collect information on how laboratory practicals, aside from anatomy, were conducted.

### 4.2 Future directions

This survey is an important step in better understanding the status and needs of new medical schools in SSA. A more comprehensive study with greater representation of new schools and possibly with interviews of key school personnel should be conducted to learn more about trends, needs, and innovations in overcoming challenges in new medical schools. These schools are a gateway to the next generation of medical doctors in SSA.

## Figures and Tables

**Figure:**
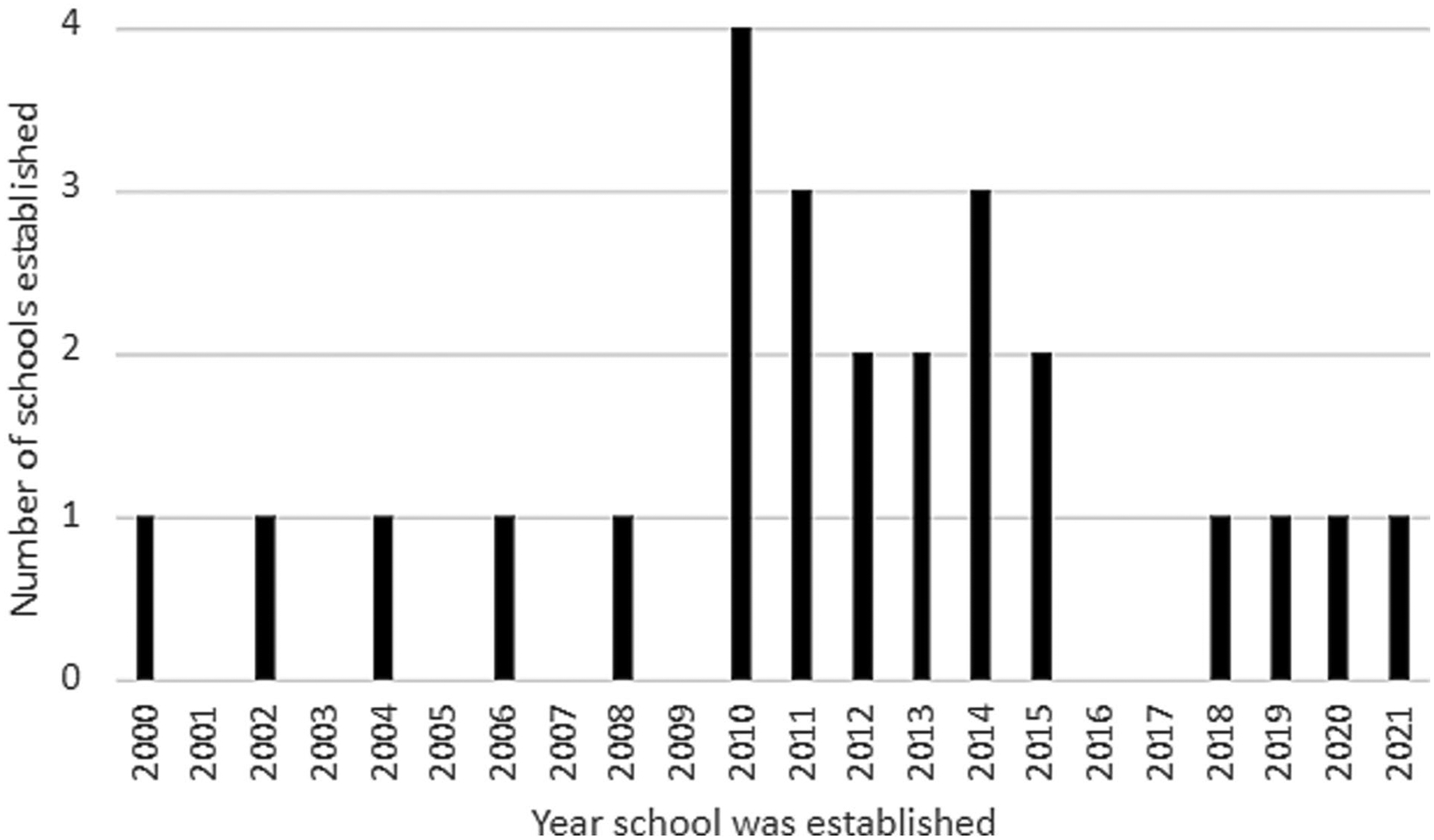
FIGURE 1 Approximate year of establishment of new medical schools in survey, 2000–2021.

**TABLE 1:** Demographics and institutional characteristics of new medical schools in Sub-Saharan Africa that responded to the survey.

Characteristics	Percentage
*Type of school (n = 25)*	
Public	44%
Private	36%
Public-Private Partnership	16%
*Location (n = 25)*	
Metropolitan Area	68%
Town	28%
Rural Area	4%
*Degree offered (n = 25)*	
MBBS	28%
MBChB	28%
MD	40%
*Length of degree (n = 24)*	
5 years	17%
6 years	63%
7 years	21%
*Gender of student population (n = 24)*	
Female	49%
Male	51%
*Funding (n = 25)*	
Government financial support	44%
Private funds	44%
Philanthropic donations	8%
Parastatal funds	4%
*Formal agreements/working relationships (n = 23)*	
National/domestic	76%
International and intercontinental (within Africa)	67%
International (outside of Africa)	62%

**TABLE 2:** Curricular components and characteristics of new medical schools in Africa that responded to the survey.

Components	Percentage
Curriculum structure (*n* = 24)	
Traditional^a^	50%
Inverted Triangle^b^	33%
Integrated^c^	17%
Origin of curriculum (*n* = 24)	
Internally developed	75%
Donated	21%
Unsure	4%
Teaching methods (*n* = 24)	
Lecture-based learning	75%
Case-based learning	63%
Problem-based learning	63%
Team-based learning	38%
Flipped-classroom learning	13%
Curricular utilization of outside learning resources (*n* = 24)	
Free, online learning resources	79%
Paid, online learning resources	17%
Other	13%
Technology used for coursework (*n* = 24)	
Information Technology (library)	84%
Laptops, tablets	75%
Smart phone applications	63%
Moodle/Blackboard	54%
Internet-based coursework	36%
Virtual reality labs/simulation labs	29%
Other	4%
Postgraduate clinical programs (*n* = 23)	
Obstetrics and Gynecology	26%
Surgery	26%
Internal Medicine	17%
Occupational/Public Health Medicine	17%
Pediatrics	17%
Anesthesia	9%
Family medicine	4%
Psychiatry	4%
None of the above	57%

a Traditional curriculum (2–3 years of biomedical science, followed by 2–3 years of clinical training).

b Inverted triangles curriculum (begins predominantly with biomedical science and gradually transitions to a more clinical focus).

c Integrated curriculum (mixture of biomedical science and clinical training throughout all/most years).

**TABLE 3:** General challenges faced, and impact of the COVID-19 pandemic reported by new medical schools in Africa that responded to the survey.

Elements	Percentage (*n* = 23)
*Challenges*	
Financial	87%
Staffing	65%
Training faculty	65%
Professional development	65%
Dependence on external resources	30%
Dependence on expatriate expertise or staff	26%
Curriculum development	22%
*Continued classes during pandemic*	
Yes	83%
No	13%
Unsure	4%
*Course delivery method during pandemic*	
In-person	22%
Online	9%
Hybrid	70%

## Data Availability

The raw data supporting the conclusions of this article will be made available by the authors, without undue reservation.

## Footnotes

1
https://wfme.org/
